# The Influence of Erbium Laser Pretreatment on Dentin Shear Bond Strength and Bond Failure Modes: A Systematic Review and Network Meta-Analysis

**DOI:** 10.3290/j.jad.b5378611

**Published:** 2024-05-24

**Authors:** Jun Wang, Shuomin Chen, Yutian Wu, Qinhui Zhang, Menghan Wu, Yuge Chen, Liang Chen, Xinhua Hong, Yilin Wang, Shengbin Huang

**Affiliations:** a Postgraduate Student, Department of Prosthodontics, School and Hospital of Stomatology, Wenzhou Medical University, Wenzhou, China. Methodology, formal analysis, data curation, wrote original draft.; b Postgraduate Student, Department of Prosthodontics, School and Hospital of Stomatology, Wenzhou Medical University, Wenzhou, China. Data curation and analysis, edited the manuscript.; c Postgraduate Student, Department of Prosthodontics, School and Hospital of Stomatology, Wenzhou Medical University, Wenzhou, China. Data curation and analysis.; d Postgraduate Student, Institute of Stomatology, School and Hospital of Stomatology, Wenzhou Medical University, Wenzhou, China. Data curation and analysis.; e Professor, Department of Prosthodontics, School and Hospital of Stomatology, Wenzhou Medical University, Wenzhou, China. Conceptualisation, supervision, project administration, proofread the manuscript, checked data of included studies.; * These authors contributed equally to this work.

**Keywords:** dentin, shear bond strength, bond failure mode, erbium laser

## Abstract

**Purpose:**

To systematically review in-vitro studies that evaluated the influence of erbium laser pretreatment on dentin shear bond strength (SBS) and bond failure modes.

**Materials and Methods:**

Electronic databases (PubMed, Cochrane Central, Embase, and Web of Science) were searched. Only in-vitro studies involving erbium laser irradiation of the dentin surface and SBS testing of the bonded resin block were included. The three common modes of bond failure (1. adhesive, 2. cohesive, and 3. mixed) were observed and analyzed. The network meta-analysis (NMA) was performed by Stata 15.0 software, the risk of bias was evaluated, and the certainty of the evidence was assessed by the Confidence in Network Meta-analysis (CINeMA).

**Results:**

Forty studies with nine pretreatments (1. blank group: BL; 2. phosphoric acid etch-and-rinse: ER; 3. self-etch adhesive: SE; 4. Er:YAG laser: EL; 5. Er,Cr:YSGG laser: ECL; 6. ER+EL; 7. ER+ECL; 8. SE+EL; 9. SE+ECL) were included in this analysis. The NMA of SBS showed that ER+EL [SMD = 0.32, 95% CI (0.11, 0.98)] had the highest SBS next to ER, especially when using one of the 3M ESPE adhesives, followed by EL, ECL, SE and SE+EL. The Ivoclar Vivadent adhesives significantly increased the SBS of the ECL [SMD = 0.37, 95% CI (0.16,0.90)] and was higher than ER+EL [SMD = 0.25,95% CI (0.07,0.85)]. Finally, the surface under the cumulative ranking curve (SUCRA) value indicated that ER+EL (SUCRA = 71.0%) and EL (SUCRA = 62.9%) were the best treatments for enhancing dentin SBS besides ER. ER+EL (SUCRA = 85.3%), ER (SUCRA = 83.7%) and ER (SUCRA = 84.3%) had the highest probability of occurring in adhesive, cohesive and mixed failure modes, respectively.

**Conclusion:**

Er:YAG and Er,Cr:YSGG lasers improved dentin SBS compared to the blank group, especially when the acid etch-and-rinse pretreatment was combined with Er:YAG laser. Shear bond strength and failure mode do not appear to be directly related.

In recent years, restorative dentistry treatment with the diagnosis and treatment of dental defects as the core has been rapidly developed, in which the dentin bonding technique has made a major contribution toward success in restorative dentistry.^42^ The smear layer formed during clinical preparation can affect dentin bonding. The traditional acid-etching technique can effectively remove the smear layer. However, there are some disadvantages, such as complicated operation steps, high technical sensitivity, and postoperative discomfort for patients, so the search for an ideal dentin surface treatment has become an important issue in the field of conserving dental tissue and restoration.^57^

Studies have pointed to the great promise of lasers in removing the smear layer and increasing the adhesive properties of dentin.^82^ The use of lasers in clinical dentistry has been highly advantageous due to their ability to irradiate the dentin without causing pain and their minimal generation of vibration or thermal effects during operation.^40^ Among many lasers, the erbium (mainly Er:YAG laser [2940 nm] and Er,Cr:YSGG lasers [2780 nm]) have a wavelength close to the peak absorption of water (3.0 μm) and hydroxyapatite (2.8 μm) in hard dental tissues; when irradiating the tooth surface, the water and hydroxyapatite therein rapidly absorb energy, causing an increase in surface temperature. The water molecules vaporize and expand, resulting in a “micro-explosion”, opening the dentin tubules and improving dentin adhesive properties.^68^ Lasers have shown unique advantages in combination with or as an alternative to traditional methods due to their high safety and precisely controlled properties.^52^

However, up to now, the literature shows no consensus on whether erbium laser pretreatment of dentin can enhance the SBS. Some studies have shown that erbium laser pretreatment of dentin enhances its bond strength,^9,51,83^ but others have reached opposing conclusions.^50,72^ In addition, the correlation between dentin bond failure modes and SBS is widely acknowledged, with bond failure modes being a significant indicator for evaluating bonding performance.^11^ The three common modes of bond failure are adhesive, cohesive, and mixed failure. Almutairi et al^8^ proposed that the incidence of cohesive failure increases as bond strength increases. Garbui et al^32^ concluded that adhesive failure frequently occurs in groups with lower bond strength. However, there has been no comprehensive report on the specific impact of laser or laser combined with conventional acid-etching pretreatment of dentin on the mode of bond failure. Certain correlations and contradictions in the results obtained from different studies may be related to different study designs, investigator expertise, and operational factors. Therefore, a systematic review and NMA is necessary to assess erbium laser’s influence on dentin SBS and bond failure modes.

With the rise of evidence-based medicine, systematic evaluation and NMA have become accepted for the objective evaluation and synthesis of research evidence for a particular problem and are usually considered the highest evidence level.^48^ NMA has been acknowledged as a reliable and robust method of generating high-quality evidence that can effectively assess the effects of multiple pretreatments. NMA can provide a ranking of optimal pretreatment strategies^55^ by evaluating direct comparisons and calculating the indirect effect size based on logical relationships.^56^ This enables clinicians and researchers to gain a more comprehensive understanding of the treatment landscape and make informed decisions about the most effective pretreatments for specific patient populations.

This study was conducted to systematically review and perform an NMA to qualitatively and quantitatively assess all published research. The purpose of this study was to analyze the effects of erbium laser on dentin bond strength and bond failure modes, and to classify and discuss different adhesives, in order to provide a theoretical basis for the clinical application of erbium laser in dentin bonding.

## Material and Methods

### Registration

A comprehensive systematic review and NMA were conducted following the guidelines outlined in the Preferred Reporting Items for Systematic Review and Meta-analysis extension statement, specifically designed for NMA studies (PRISMA-NMA).^54^ The protocol was registered with the International Prospective Register of Systematic Reviews (PROSPERO) under the unique registration identifier CRD42023399845.

### Search Strategy

An extensive electronic literature search was conducted across multiple databases, including Medline via PubMed, Cochrane Central, Embase, and Web of Science. The search encompassed a wide range of publication years with no restrictions. The search period extended from September 10, 2022, to June 19, 2023. The search strategies employed three key domains: erbium laser, dentin, SBS. The search strategy was formulated as follows:

- #1: Erbium laser OR erbium YAG laser OR erbium-doped laser OR erbium-based laser OR erbium-ion laser OR erbium-doped fiber laser
- #2: Er:YAG OR erbium doped:yttrium aluminum garnet
- #3: Er,Cr:YSGG OR erbium,chromium:yttrium scandium gallium garnet
- #4: #1 OR #2 OR #3
- #5: Preteatment OR treatment OR preparation OR prepping OR etching OR modification OR irradiation OR ablation OR therapy
- #6: Dentin* AND strength AND bond*[MESH]
- #7: #4 AND #5 AND #6

Additionally, a manual search was conducted by reviewing the reference lists of included studies that were not identified through the electronic search.

### Eligibility Criteria

The NMA included studies that met the following inclusion criteria: (i) full-text articles or theses published in English; (ii) online publications before June 19, 2023; (iii) assessment of dentin SBS using various treatments, including a control group, erbium laser treatment (EL, ECL), acid etching treatment (ER, SE), and combinations of laser and acid etching treatment (ER+EL, ER+ECL, SE+EL, SE+ECL); (iv) inclusion of at least one mode of laser; (v) inclusion of SBS data (MPa) or the modes of bonding failure.

### Exclusion Criteria

Studies or groups meeting one or more of the following criteria were excluded: (i) non in-vitro studies, reviews, systematic evaluations, observational studies, etc; (ii) studies lacking quantitative data on standard mean deviation (SMD) of SBS; (iii) duplicate reporting of the same bond strength data in multiple publications; (iv) Er:YAG laser or Er,Cr:YSGG laser was not included; (v) use of tooth modes other than isolated human permanent teeth or non-dentin samples; (vi) studies that performed microshear bond strength tests. Due to the limited number of studies including microshear bond strength yielded by our literature screening process, we decided to focus on studies that assess shear bond strength, not microshear bond strength, as the outcome measure to ensure the reliability and accuracy of this research.

Access to partially missing data from potentially included studies was gained by reaching out to the respective authors. If the respective author could not provide the necessary information, studies providing insufficient data were excluded.

### Screening and Selection

The studies identified during the search process were imported into EndNote 20 software to eliminate duplicate entries. Two researchers (J.W. and S.C.) independently evaluated the screened and selected studies. Any discrepancies in the selection were resolved through mutual consultation and discussion.

### Data Extraction and Collection

The data from the studies included in the analysis were independently extracted by two researchers (J.W. and S.C.). To facilitate data collection, Microsoft Excel spreadsheets (Microsoft; Redmond, WA, USA) were utilized. The dental pretreatment measures were divided into nine categories: (i) blank group (BL) (neither erbium laser treatment nor acid etching treatment); (ii) acid etch-and-rinse (ER); (iii) self-etch adhesive (SE); (iv) Er:YAG laser (EL); (v) Er,Cr:YSGG laser (ECL); (vi) acid etch-and-rinse combined with Er:YAG laser (ER+EL); (vii) acid etch-and-rinse combined with Er,Cr:YSGG (ER+ECL); (viii) self-etch adhesive combined with Er:YAG laser (SE+EL); (ix) self-etch adhesive combined with Er,Cr:YSGG (SE+ECL).

### Risk of Bias

We used a scoring system comprising seven rating criteria to assess the risk of bias in the studies included. The parameters used to assess risk of bias were revised and adapted in accordance with previous research^62^ and carried out according to the description of the followoing study quality-assessment parameters: (i) sample size calculation); (ii) randomization of teeth or specimens); (iii) specimens with similar dimensions); (iv) lasers used according to manufacturer’s instructions); (v) adhesive procedures conducted by the same operator); (vi) operator blinding in the testing machine); (vii) evaluation of failure mode. Each study was assigned a “yes” score if it reported the item and a “no” score if no information was given. Studies reporting three or fewer items were considered to have a high overall risk of bias. In comparison, those reporting four or five items were classified as having a medium risk, and those reporting six or seven items were deemed to have a low risk of bias. The quality and risk of bias of each study were evaluated independently by two reviewers (J.W. and S.C.) and disagreements were resolved through discussion.

### Statistical Analysis

In our study, we conducted an NMA using Stata software version 15.0.^59,65,77^ We calculated the summary SMD along with a corresponding 95% confidence interval (CI) for continuous data. In contrast, we computed the summary odds ratio (OR) with a 95% CI for categorical data. Statistical significance was defined as p-values less than 0.05 (p<0.05).^17^ To visually illustrate direct comparisons between different pretreatments, we utilized a network diagram in which node size represented the sample size of each pretreatment, and the thickness of the connecting lines indicated the number of studies directly comparing the two pretreatments.^45^ We then evaluated both global and local inconsistency through the node-splitting method to determine whether the estimated effects from direct and indirect comparisons were consistent. If p > 0.05, we employed the consistency model; otherwise, we used the inconsistency model. To rank the SBS and bond failure modes of the pretreatments, we calculated the probabilities of the surface under the cumulative ranking curve (SUCRA) between all pretreatments. We also created league tables summarizing the outcomes of each indicator through both direct and indirect comparisons. We used SUCRA to estimate the dentin SBS of different treatments. As a simple numerical summary that complements graphical displays of each pretreatment, SUCRA offers the advantage of simplifying information on the effects of each pretreatment into a few numbers.^61^ The values of SUCRA range from 0 to 100%, with higher values indicating more effective treatment and lower values indicating less effective treatments.^60^ We use the chi-squared test to explore the potential heterogeneity in the sources of the articles.^19^ If heterogeneity existed, we analyzed the possible sources of heterogeneity. In addition, funnel plots and Egger’s test were used to detect publication bias in the articles. A more symmetrical distribution of scatter points with the same color on both sides of the funnel plot indicate a smaller publication bias, and the linear regression lines spanning both sides of the funnel plot visually represents the publication bias between different articles.

Finally, we categorized the adhesives of 40 articles into 4 groups: 3M ESPE (17 papers), Ivoclar Vivadent (12 papers), Kuraray Dental (4 papers), and others (7 papers). We then conducted a Network Meta-Analysis (NMA) to analyze the impact of different adhesives on bond strength under various adhesive methods.

### Quality of Evidence

We used an online quality assessment software, “Confidence In Network Meta-Analysis (CINeMA)”, which is based on the Grading of Recommendations Assessment, Development and Evaluation (GRADE) framework, to grade the quality of evidence in NMA.^53^ Unlike the standard GRADE methodology, which recommends separate grading for direct evidence, indirect evidence, and NMA evidence,^38^ CINeMA considers NMA as a whole and assesses the quality of NMA evidence based on the integration of six domains: risk of bias within studies, publication bias or reporting bias, indirectness, imprecision, heterogeneity, and incoherence. For each domain, the severity can be categorized as not serious (no concern, no downgrade), serious (some concern, one level downgrade), or very serious (major concern, two levels downgrade). Ultimately, the quality of NMA evidence is classified as high, moderate, low, or very low.

## Results

### Identifying Studies

**Fig 1:**
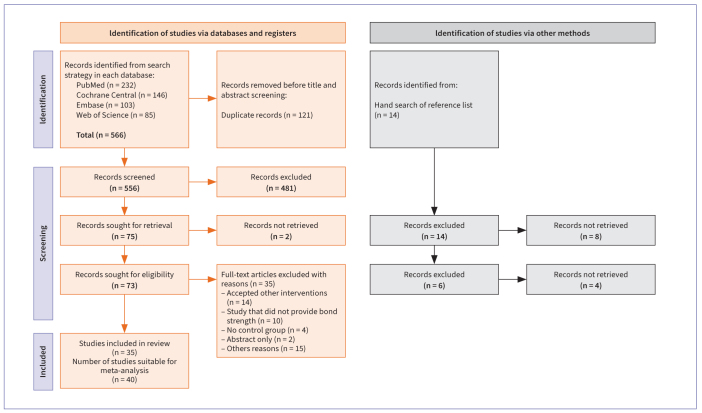
Flowchart detailing search strategy.

Based on a preliminary electronic search, 687 articles were retrieved (Fig 1). After removing duplicates, 566 articles remained. Upon reviewing the titles and abstracts, 72 articles met the criteria for full-text reading. After thoroughly examining the full texts, 40 studies were ultimately included.

### Study Characteristics

The NMA identified 40 studies that reported single or combined treatments with EL, ER, SE, EL, and ECL. These studies were published over the period of 1996 to 2023, involving a total of 1450 dental samples. Twenty-one studies reported SBS and bond failure modes, while the remaining 19 studies reported only SBS. Table 1 shows the details of 40 studies.

**Table 1:** Characteristics of the included studies

Authors, year	Country	Tooth type	No of specimens	Surface treatment	Shear bond strength (MPa) Mean ±SD	Numbers of failure mode (adhesive, cohesive, mixed)	Laser application parameters	Adhesive	Irradiation distance and duration	Spot size of laser beam	Water cooling	Speed of SBS test machine (mm/min)
Al Habdan et al 2021^3^	Saudi Arabia	molars	10	ER ECL	16.25 ± 1.10 8.56 ± 0.67	(8, 2, 0) (10, 0, 0)	4.50 W, 50 Hz, 90 mJ	Ivoclar Vivadent: ExciTE F	0.5 mm/20 s	0.8 mm	30% water, 60% air	0.5
Al-Jeaidi et al 2020^1^	Saudi Arabia	third molars	10	ER TE+ECL	19.21 ± 0.93 16.13 ± 3.01	(2, 3, 10) (1, 6, 8)	4.50 W, 30 Hz					
Alkhudhairy et al 2019(1)^5^	Saudi Arabia	molars	15	SE SE+EL	18.96 ± 0.32 7.48 ± 1.31	(6, 2, 7) (6, 0, 9)	4.50 W, 30 Hz					
Alkhudhairy et al 2019(2)^6^	Saudi Arabia	molars	15	SE ECL	18.45 ± 1.34 18.31 ± 1.17	(12, 3, 0) (5, 0, 10)	4.50 W, 30 Hz					
Al-khureif et al 2020^2^	Saudi Arabia	molars	10	ER ECL	23.15 ± 3.21 17.44 ± 2.77	(1, 6, 3) (1, 2, 7)	0.50 W, 30 Hz	Others	2 mm/60 s		constant air/water	0.5
Almutairi et al 2021^8^	Saudi Arabia	molars	20	ER ECL	17.84 ± 0.93 18.31 ± 0.25	(4, 12, 4) (6, 2, 12)	4.50 W, 30 Hz	KaVo Kerr: All-In-One	2 mm/60 s		1.5 ml/min water	1
Altunsoy et al 2014^9^	Turkey	molars	19	BL ER EL	2.53 ± 1.12 6.61 ± 1.99 3.24 ± 1.03	(10, 0, 0) (5, 2, 3) (9, 1, 0)	10 Hz, 120 mJ					
Bahrololoomi et al 2017^13^	Iran	molars	14	BL EL	13.56 ± 3.36 20.33 ± 4.82		200 mJ, 10 Hz	Bisco: One-Step Plus bonding	17 mm		constant air/water	0.5
Beer et al 2011^14^	Austria	molars	10	ER SE EL ER+EL SE+EL	13.03 ± 3.66 14.86 ± 3.66 14.07 ± 2.11 9.65 ± 2.11 14.07 ± 2.11		2.00 W, 13.4 J/cm^2^	Ivoclar Vivadent: Syntac Classic			55% water, 65% air	1
Bertrand et al 2006^16^	France	molars	15	BL EL ER+EL	16.74 ± 6.24 20.33 ± 4.82 17.59 ± 5.96	(4, 3, 8) (2, 1, 12) (3, 1, 11)	10 Hz, 500 mJ	Ivoclar Vivadent: Astralis 5	400 μs	1.2 mm	constant air/water	3
Brulat et al 2008^18^	France	third molars	20	SE SE+EL	13.87 ± 5.25 9.62 ± 3.28		10 Hz, 500 mJ	Kuraray Dental: Clearfil SE Bond; two bottles	12 mm/15 s	0.8 mm	constant air/water	1.2
Capa et al 2010^20^	Turkey	third molars	10	BL TE+EL	4.92 ± 1.68 8.79 ± 2.98		30 Hz, 70.0 mJ	3M ESPE: SmartCem2, RelyX Unicem, Multilink Automix	100 ms	600 mm	constant air/water	0.5
Ceballo et al 2002^21^	Spain	third molars	20	ER EL ER+EL	22.50 ± 3.40 4.00 ± 2.20 16.70 ± 2.90		2Hz, 180 mJ	3M ESPE: Single Bond	20 mm/250 ms	1 mm	constant air/water flow	0.75
Chemaly et al 2022^22^	Lebanon	third molars	12	SE EL ER+EL	13.38 ± 8.53 3.00 ± 2.20 16.70 ± 2.90		4.50 W, 50 Hz, 90 mJ	3M ESPE: RelyX Ultimate Clicker, 3M ESPE	1.5 mm/60 μs	800 μm	80% water, 40% air	1
Chou et al 2009^23^	China	third molars	5	ER ECL	19.06 ± 4.06 15.53 ± 5.27		5.00 W, 20 Hz	3M ESPE: Single Bond 2	1 mm/140 ms	600 mm	50% water, 70% air	0.5
Curylofo et al 2014^24^	Brazil	molars	10	SE SE+EL	5.66 ± 1.77 7.48 ± 1.31	(3, 2, 5) (2, 2, 6)	15 Hz, 400 mJ					
Cvikl et al 2011^25^	Austria	third molars	15	BL ECL	3.93 ± 1.55 7.37 ± 4.44		2.00 W, 30 Hz	Ivoclar Vivadent: Variolink II/Syntac adhesive	1 mm	600 μm	constant air/water	0.8
Dilber et al 2015^28^	Turkey	central incisor	20	BL ER EL ER+EL	2.01 ± 1.51 4.61 ± 1.47 5.45 ± 1.07 5.16 ±2.69	(2, 0, 11) (5, 0, 4) (7, 1, 5) (10, 0, 2)	10Hz, 120 mJ					
Dunn et al 2005^29^	USA	molars	20	BL ER EL ER+EL	3.40 ± 2.10 19.80 ± 3.60 7.40 ±2.00 10.02 ± 2.80	(2, 14, 4) (4, 6, 10) (11, 3, 6) (6, 6, 8)	30 Hz, 140 mJ	3M ESPE: Adper Scotchbond Multi-Purpose	1 mm	0.6 mm	5 ml/min water	0.5
Elsahn et al 2021^30^	UAE	molars	12	ER SE ER SE+E;	23.20 ± 6.80 25.48 ± 2.60 17.73 ± 2.69 12.22 ± 3.00	(4, 6, 0) (8, 2, 0) (8, 2, 0) (8, 2, 0)	4.00 W, 20Hz, 200 mJ					
Garbui et al 2013^32^	Brazil	molars	17	BL ECL	4.79 ± 0.82 10.56 ± 1.16	(33, 11, 1) (22, 21, 2)	0.50 W, 20 Hz, 25 mJ	Others			75% water, 85% air	1
Giray et al 2014^33^	Turkey	molars	10	ER ECL ER+ECL	10.71 ± 5.47 6.34 ± 1.35 8.14 ± 1.69	(2, 6, 2) (5, 1, 4) (4, 5, 1)	1.50 W, 20 Hz, 120 mJ	Ivoclar Vivadent: adhesive resin [Variolink II (V)] cement	2 mm/15 s	0.6 mm	35 ml/min water	0.5
Gisler et al 2012^34^	Switzerland	third molars	12	SE ECL SE+EL	16.70 ± 8.98 13.67 ± 6.11 17.34 ± 6.97		15 Hz, 70.0 mJ	Ivoclar Vivadent:	2 mm/8s		55% water, 65% air	1
Guven et al 2013^37^	Turkey	molars	15	BL ER EL ER+EL	13.18 ± 2.59 17.81 ±4.24 15.20 ± 4.68 17.46 ± 5.11	(2, 3, 10) (1, 6, 8) (4, 1, 10) (4, 2, 9)	200 mJ, 20 Hz					
Gurgan et al 2008^35^	Turkey	molars	10	BL ER EL ER+EL	10.53 ± 1.01 13.01 ± 2.09 11.37 ± 1.80 10.28 ± 1.94		5.00 W, 20 Hz,					
Ismatullaev 2020^39^	Turkey	molars	12	SE EL	8.17 ± 2.60 10.05 ± 4.61		10 Hz, 20.0 mJ					
Jhingan et al 2015^41^	India	molars	16	BL SE ECL SE+ECL	20.99 ± 3.65 15.94 ± 3.11 33.36 ± 9.19 21.04 ± 3.57		6.00 W, 15 Hz					
Karadas et al 2017^44^	Turkey	molars	10	SE EL	17.42 ± 4.10 28.51 ± 4.30	(9, 2, 4) (7, 2, 6)	1.20 W, 10 Hz	Kuraray Dental: Clearfil SE Bond	10 mm/150 μs	0.9 mm	80% water, 40% air	1
Meriç et al 2016^50^	Turkey	molars	15	BL ECL	23.91 ± 9.73 19.20 ± 7.71		2.00 W, 20 Hz, 100 mJ					
Nahas et al 2016^51^	Lebanon	third molars	12	SE EL	8.17 ± 2.60 10.05 ± 4.61		10 Hz, 80 mJ	Others	100 μs	1.3 mm	80% water, 40% air	1
Ribeiro et al 2013^58^	Brazil	molars	14	ER EL	17.05 ± 4.15 12.12 ± 3.85	(10, 1, 3) (11, 0, 3)	20 Hz, 60 mJ	3M ESPE: Single Bond	2 mm/30 s		1.5 ml/min water	0.5
Sharafeddin et al 2022^64^	Iran	molars	10	ER EL	13.35 ± 1.47 6.92 ± 0.90	(2, 8, 0) (5, 5, 0)	1.50 W, 10 Hz, 50 mJ	3M ESPE: Single Bond	20 mm/30 s		constant air/water	1
Shirani et al 2012^67^	Iran	premolars	10	ER EL TE+EL	12.89 ± 3.88 10.03 ± 2.56 12.01 ± 2.07	(2, 8, 0) (5, 5, 0) (2, 8, 0)	4.0 Hz, 160 mJ	3M ESPE: Single Bond	0.5 mm/40 s	0.9 mm	7 ml/min water	1
Shirani et al 2014^66^	Iran	third molars	10	SE SE+EL	14.43 ± 4.54 12.31 ± 4.90		30 Hz, 140 mJ	3M ESPE: Single Bond	0.5 mm/150 µs	2 mm	constant air/water	1
Staninec et al 2006^69^	USA	molars	10	ER EL	31.90 ± 5.0 23.40 ± 3.00			3M ESPE: Single Bond		1/2 mm	24 ml/min water	5
Ustunkol et al 2015^72^	Turkey	third molars	15	BL SE ECL	13.92 ± 8.06 34.10 ± 12.33 20.98 ± 6.68	(12, 3, 0) (2, 7, 6) (7, 7, 1)	1.25 W, 20 Hz					
Visuri et al 1996^74^	USA	molars	9	BL ER EL ER+EL	8.10 ± 4.10 7.30 ± 4.30 12.90 ± 7.30 7.10 ± 5.00		6.0 Hz, 350 mJ	Dentsply Sirona: ProBOND, Caulk/Dentsply		1 mm	constant air/water flow	2.5
Vohra et al 2018^75^	Saudi Arabia	molars	20	ER SE ER+EL SE+EL	23.06 ± 1.14 13.02 ± 1.01 23.66 ± 2.56 11.87 ± 1.21		4.50 W, 50 Hz	Harvard Dental: SE bond [Harvard Bond SE Mono]	2 mm/60 s		80% water, 40% air	1
Xiong et al 2022^79^	China	molars	10	ER ER+ECL	19.09 ± 2.66 15.19 ± 1.87		100 Hz, 3.00 mJ					
Yazici et al 2010^81^	Turkey	molars	20	SE SE+EL	17.89 ± 4.57 10.16 ± 2.14	(7, 2, 11) (13, 2, 5)	3.0 Hz, 100 mJ	Kuraray Dental: Clearfil Tri-S Bond, Kuraray Medical	6 mm/60 s	5 mm	40–60 ml/min water	1

BL: blank group; ER: etch-and-rinse; SE: self-etch adhesive; EL: Er:YAG laser; ECL: Er,Cr:YSGG laser.

### Risk of Bias

Of the 40 included studies, 11 were considered to have a low risk of bias, while 26 were considered to have a moderate risk of bias. In addition, 3 studies were identified as having a high risk of bias (Supplementary Table 1).

**Supplementary Table 1:** Risk of bias

Study	Sample size calculation	Randomization of teeth / specimens	Specimens with similar dimensions	Lasers used according to manufacturer’s instructions	Adhesive procedures conducted by the same operator	Operator blinding in the testing machine	Evaluation of failure mode	Risk of bias
Al Habdan et al 2021	no	yes	yes	yes	yes	yes	yes	low
Al-Jeaidi et al 2020	no	yes	yes	yes	yes	no	yes	medium
Alkhudhairy et al 2019(1)	no	yes	yes	yes	yes	no	yes	medium
Alkhudhairy et al 2019(2)	no	no	yes	yes	yes	yes	yes	low
Al-khureif et al. 2020	no	yes	yes	yes	no	no	yes	medium
Almutairi et al 2021	no	yes	yes	yes	no	no	yes	medium
Altunsoy et al 2014	no	yes	yes	yes	no	no	yes	medium
Bahrololoomi et al 2017	no	yes	yes	yes	no	no	no	medium
Beer et al 2011	yes	yes	yes	yes	yes	yes	no	low
Bertrand et al 2006	no	yes	yes	yes	yes	yes	yes	low
Brulat 2008	no	yes	yes	yes	no	no	no	medium
Capa et al 2010	no	yes	yes	yes	no	no	no	medium
Ceballo et al 2002	no	no	yes	yes	no	no	no	high
Chemaly et al 2022	no	yes	yes	yes	yes	no	no	medium
Chou et al 2009	no	yes	yes	yes	yes	no	no	medium
Curylofo et al 2014	yes	yes	yes	yes	no	no	yes	medium
Cvikl et al 2011	yes	yes	yes	yes	yes	yes	no	low
Dilber et al 2015	yes	yes	yes	yes	yes	yes	no	low
Dunn et al 2005	no	yes	yes	yes	yes	yes	yes	low
Elsahn et al 2021	yes	no	yes	yes	no	no	yes	medium
Garbui et al 2013	no	yes	yes	yes	no	no	yes	medium
Giray et al 2014	no	no	yes	yes	yes	yes	yes	low
Gisler et al 2012	no	yes	yes	yes	no	yes	no	medium
Guven et al 2013	no	no	yes	yes	no	no	yes	medium
Gurgan et al 2008	yes	yes	yes	yes	no	no	no	medium
Ismatullaev et al 2020	yes	yes	yes	yes	yes	yes	no	low
Jhingan et al 2015	no	no	yes	yes	no	no	no	medium
Karadas et al 2017	no	yes	yes	yes	no	no	yes	medium
Meriç et al 2016	no	yes	yes	yes	yes	no	no	medium
Nahas et al 2016	no	no	yes	yes	no	yes	no	medium
Ribeiro et al 2013	yes	no	yes	yes	no	no	yes	medium
Sharafeddin et al 2022	no	yes	yes	yes	yes	yes	yes	low
Shirani et al 2012	no	yes	yes	yes	no	no	yes	medium
Shirani et al 2014	yes	yes	yes	yes	no	no	no	medium
Staninec et al 2006	yes	yes	yes	yes	no	no	no	medium
Ustunkol et al 2015	no	no	yes	yes	no	no	no	high
Visuri et al 1996	yes	yes	yes	yes	yes	yes	no	low
Vohra et al 2018	yes	yes	yes	yes	no	no	no	medium
Xiong et al 2022	no	no	yes	yes	no	no	no	high
Yazici 2010	no	yes	yes	yes	no	no	yes	medium

If the parameter was mentioned in the text and the study received a “yes” for that specific parameter, it was classified as “yes”. Otherwise, it was classified as “no”. The risk of bias was categorized based on the total number of “yes” responses as follows: 1-2 = high, 3-4 = medium, 5-6 = low risk of bias.

### Network Plot

**Fig 2:**
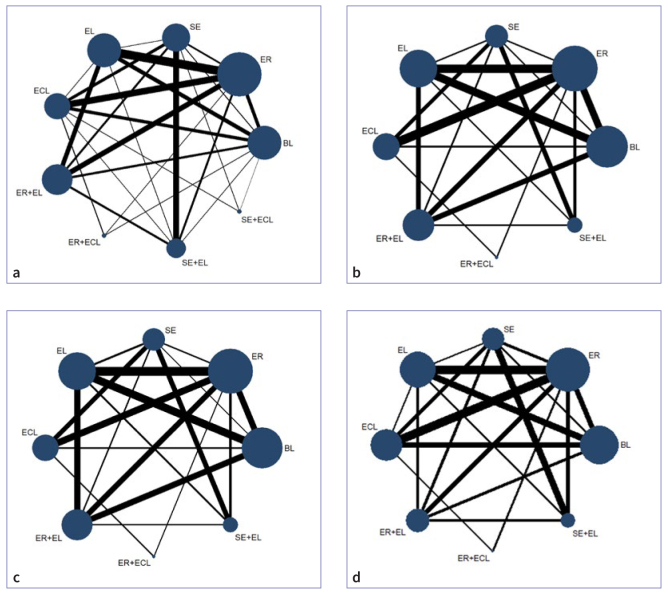
The network plots for different pretreatments. a. bond strength; b. adhesive failure mode; c. cohesive failure mode; d. mixed failure mode.

Dentin shear bond strength consists of nine pretreatments (BL, ER, SE, EL, ECL, ER+EL, ER+ECL, SE+EL, SE+ECL), and adhesive, cohesive, and mixed failure modes consist of eight pretreatments (BL, ER, SE, EL, ECL, ER+EL, ER+ECL, SE+EL). Twenty-one of the 50 studies reported both SBS and bond failure modes (Fig 2a), while the remaining 19 studies reported only SBS (Fig 2b–d). In the figure, the lines indicate direct comparisons between pretreatments. The thicker the line, the more studies have been conducted on that comparison. Additionally, the size of the dots represents the sample size used in those studies.

### Inconsistency Test

**Supplementary Fig 1:**
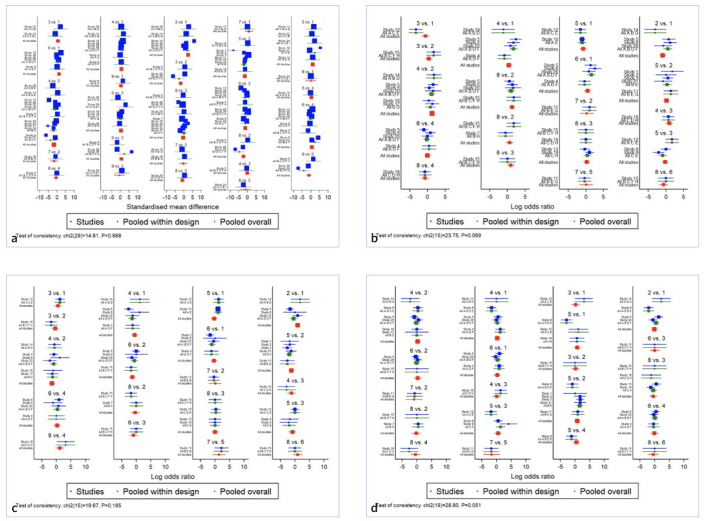
Forest plot and the global inconsistency test result. a. bond strength chi^2^ = 14.81, p = 0.988; b. adhesive failure mode chi^2^ = 23.75, p = 0.069; c. cohesive failure mode chi^2^ = 19.67, p = 0.185; d. mixed failure mode chi^2^ = 28.80, p = 0.051). BL = 1 = A, ER = 2 = B, SE = 3 = C, Er:YAG = 4 = D, Er,Cr:YSGG = 5 = E, ER+Er:YAG = 6 = F, ER+Er,Cr:YSGG = 7 = G, SE+Er:YAG = 8 = H, SE+ER,Cr:YSGG = 9 = I.

The outcomes of the global inconsistency tests, including SBS and failure modes (adhesive, cohesive, and mixed), are depicted in Supplementary Fig 1a–d). Moreover, the findings from the local inconsistency tests involving SBS and the 3 failure modes are presented in Supplementary Tables 2 to 5.

**Supplementary Table 2:** Local inconsistency test (shear bond strength)

Side	Direct		Indirect		Difference		p>|z|
	Coefficient	SE	Coefficient	SE	Coefficient	SE	
BL vs ER	2.055	0.778	2.924	0.761	-0.868	1.088	0.425
BL vs SE	0.600	1.343	0.693	0.776	-0.093	1.552	0.952
BL vs EL	0.770	0.671	0.665	1.079	0.105	1.270	0.934
BL vs ECL	1.746	0.775	0.350	0.866	1.396	1.163	0.230
BL vs ER+EL	0.637	0.839	2.278	0.931	-1.641	1.253	0.190
BL vs ER+ECL	0.214	1.889	1.458	1.606	-1.244	2.479	0.616
BL vs SE+EL	1.532	1.921	0.164	0.799	1.368	2.081	0.511
BL vs SE+ECL	0.009	1.890	0.556	2.921	-0.547	3.479	0.875
ER vs SE	-1.460	1.107	-2.010	0.773	0.549	1.344	0.683
ER vs EL	-1.614	0.533	-2.682	1.340	1.069	1.441	0.459
ER vs ECL	-1.404	0.697	-1.337	0.835	-0.067	1.087	0.951
ER vs ER+EL	-1.034	0.634	-1.498	1.277	0.464	1.427	0.745
ER vs ER+ECL	-0.728	1.909	-2.133	1.573	1.405	2.475	0.570
ER vs SE+EL	-2.786	1.110	-1.704	0.902	-1.082	1.425	0.448
SE vs EL	-0.269	1.910	0.124	0.762	-0.393	2.056	0.848
SE vs ECL	0.290	0.775	0.721	0.986	-0.431	1.254	0.731
SE vs TE+EL	0.788	1.114	0.648	0.926	0.140	1.444	0.923
SE vs TE+ECL	-0.345	0.604	0.044	1.764	-0.389	1.864	0.835
SE vs SE+EL	0.913	1.853	-3.930	2.892	4.843	3.442	0.159
SE vs SE+ECL	-0.159	1.891	0.455	0.682	-0.614	2.010	0.760
EL vs ECL	0.325	0.669	1.700	1.245	-1.375	1.414	0.331
EL vs ER+EL	0.000	1.910	-0.447	0.837	0.447	2.085	0.830
EL vs ER+ECL	-0.238	1.346	-0.012	2.560	-0.226	2.895	0.938
ECL vs ER+ECL	0.481	1.893	-0.965	0.772	1.446	2.044	0.479
ECL vs SE+EL	-2.229	1.873	1.944	2.837	-4.173	3.417	0.222
ECL vs SE+ECL	-2.112	1.098	-0.037	1.026	-2.074	1.497	0.166
ER+EL vs ER+ECL	2.055	0.778	2.924	0.761	-0.868	1.088	0.425
ER+EL vs SE+EL	0.600	1.343	0.693	0.776	-0.093	1.552	0.952

BL: blank group; ER: etch-and-rinse; SE: self-etch adhesive; EL: Er:YAG laser; ECL: Er,Cr:YSGG laser.

**Supplementary Table 3:** Local inconsistency test (adhesive failure mode)

Side	Direct		Indirect		Difference		p>|z|
	Coefficient	SE	Coefficient	SE	Coefficient	SE	
BL vs ER	-0.029	0.593	-1.759	0.592	1.729	0.830	0.037
BL vs SE	-3.185	1.094	0.190	0.579	-3.375	1.239	0.006
BL vs EL	0.830	0.510	-0.860	0.887	1.691	1.016	0.096
BL vs ECL	-1.159	0.616	-0.223	0.710	-0.936	0.938	0.318
BL vs ER+EL	0.934	0.530	-1.657	1.102	2.591	1.240	0.037
TE vs SE	1.807	1.171	0.058	0.548	1.749	1.292	0.176
TE vs EL	1.230	0.453	1.582	0.866	-0.351	0.980	0.720
TE vs ECL	0.602	0.570	-0.442	0.632	1.045	0.851	0.220
TE vs ER+EL	1.280	0.553	1.491	0.939	-0.211	1.096	0.847
TE vs ER+ECL	0.981	1.181	-1.903	2.132	2.883	2.522	0.253
TE vs SE+EL	0.679	0.863	0.582	0.746	0.097	1.136	0.932
SE vs EL	0.690	1.065	1.021	0.640	-0.331	1.241	0.790
SE vs ECL	-0.242	0.612	-0.210	0.819	-0.031	1.017	0.976
SE vs ER+EL	0.000	1.284	1.221	0.670	-1.221	1.448	0.399
SE vs SE+EL	0.248	0.518	0.270	1.341	-0.023	1.438	0.987
EL vs ER+EL	-0.035	0.514	0.292	1.048	-0.328	1.166	0.779
EL vs SE+EL	-0.699	1.067	-0.676	0.723	-0.024	1.288	0.985
ECL vs ER+ECL	-0.405	1.082	2.478	2.285	-2.883	2.522	0.253
ER+EL vs SE+EL	0.000	1.289	-0.946	0.748	0.946	1.490	0.525

BL: blank group; ER: etch-and-rinse; SE: self-etch adhesive; EL: Er:YAG laser; ECL: Er,Cr:YSGG laser.

**Supplementary Table 4:** Local inconsistency test (cohesive failure mode)

Side	Direct		Indirect		Difference		p>|z|
	Coefficient	SE	Coefficient	SE	Coefficient	SE	
BL vs ER	-0.319	0.539	2.401	0.571	-2.72086	0.804855	0.001
BL vs SE	1.160	1.163	0.279	0.824	0.881256	1.428916	0.537
BL vs EL	-1.130	0.621	1.055	1.138	-2.18461	1.324778	0.099
BL vs ECL	1.051	0.400	-2.310	0.624	3.36098	0.740519	0
BL vs ER+EL	-1.141	0.590	2.379	1.106	-3.5197	1.253405	0.005
TE vs SE	-1.822	1.288	0.042	0.697	-1.86424	1.462205	0.202
TE vs EL	-0.949	0.504	-4.332	1.062	3.382809	1.163894	0.004
TE vs ECL	-2.016	0.534	0.245	0.692	-2.2604	0.874856	0.01
TE vs ER+EL	-0.926	0.630	-2.568	1.157	1.641841	1.304265	0.208
TE vs ER+ECL	-0.405	1.201	2.639	2.862	-3.0445	3.109688	0.328
TE vs SE+EL	-0.960	0.947	0.313	0.974	-1.27301	1.352258	0.347
SE vs EL	-2.233	1.643	-0.956	0.791	-1.27725	1.789756	0.475
SE vs ECL	-0.855	0.839	-0.922	0.977	0.067482	1.276906	0.958
SE vs ER+EL	0.000	1.386	-1.286	0.874	1.285521	1.638386	0.433
SE vs SE+EL	0.100381	0.648134	-0.34651	1.572931	0.44689	1.701462	0.793
EL vs ER+EL	0.2303	0.733613	0.350518	1.293764	-0.12022	1.494088	0.936
EL vs SE+EL	2.811797	1.616091	0.76001	0.876738	2.051786	1.821643	0.26
ECL vs ER+ECL	2.197225	1.46207	-0.84738	2.471927	3.044606	3.10971	0.328
ER+EL vs SE+EL	9.43E-09	1.38433	1.424982	0.971355	-1.42498	1.691124	0.399

BL: blank group; ER: etch-and-rinse; SE: self-etch adhesive; EL: Er:YAG laser; ECL: Er,Cr:YSGG laser.

**Supplementary Table 5:** Local inconsistency test (mixed failure mode)

Side	Direct		Inderict		Difference		p>|z|
	Coefficient	SE	Coefficient	SE	Coefficient	SE	
BL vs ER	0.073	0.672	-0.221	0.966	0.294	1.173	0.802
BL vs SE	3.643	1.610	-0.607	0.782	4.250	1.797	0.018
BL vs EL	0.021	0.595	1.691	1.425	-1.671	1.544	0.279
BL vs ECL	-1.241	0.762	2.249	0.662	-3.490	0.992	0.000
BL vs ER+EL	0.527	0.681	-1.178	1.526	1.704	1.676	0.309
ER vs SE	0.000	2.265	0.253	0.709	-0.253	2.374	0.915
ER vs EL	-0.255	0.495	2.510	0.991	-2.765	1.107	0.013
ER vs ECL	1.079	0.565	-0.109	0.942	1.188	1.097	0.279
ER vs ER+EL	-0.075	0.779	0.935	1.107	-1.010	1.354	0.456
ER vs ER+ECL	-0.811	1.650	-1.308	2.744	0.498	3.046	0.870
ER vs SE+EL	0.301	1.153	-0.621	1.043	0.922	1.554	0.553
SE vs EL	1.509	1.388	-0.461	0.826	1.969	1.622	0.225
SE vs ECL	0.370	0.827	0.812	1.067	-0.442	1.357	0.745
SE vs ER+EL	0.000	2.266	0.036	0.900	-0.036	2.439	0.988
SE vs SE+EL	-0.377	0.750	-0.704	1.592	0.327	1.750	0.852
EL vs ER+EL	-0.887	1.341	0.842	0.704	-1.729	1.513	0.253
EL vs SE+EL	-0.042	0.723	-0.008	1.426	-0.034	1.603	0.983
ECL vs ER+ECL	-2.793	1.704	0.185	0.908	-2.978	1.951	0.127
ER+EL vs SE+EL	-1.792	1.585	-1.294	2.856	-0.498	3.046	0.870

BL: blank group; ER: etch-and-rinse; SE: self-etch adhesive; EL: Er:YAG laser; ECL: Er,Cr:YSGG laser.

The tests for global and local inconsistency both indicate no significant discrepancy between direct and indirect comparison. Therefore, a consistency model was used to evaluate shear bond strength and the 3 failure modes.

### Network Meta-Analysis

The NMA showed that compared to the BL, ER [SMD = 12.16, 95% CI (4.22,35.07 )], ER+EL [SMD = 3.95, 95% CI (1.16,13.45)], EL [SMD = 3.20, 95% CI (2.69,6.33)] and ECL [SMD = 1.08, 95% CI (1.99,9.55)] all improved the dentin SBS. SE+EL [SMD = 0.12, 95% CI (0.03,0.47)], SE [SMD = 0.16, 95% CI (0.05,0.55)], ECL [SMD = 0.17, 95% CI (0.07,0.45)], EL [SMD = 0.25, 95% CI (0.0.9,0.72)], and ER+EL [SMD = 0.32, 95% CI (0.11,0.98)] did not as effectively enhance dentin SBS as did the ER treatment (Table 2).

**Table 2:** Standardized mean differences (SMDs) and 95% CI of bond strength of dentin

SE+ECL								
0.82 (0.03,20.14)	SE+EL							
0.47 (0.01,20.24)	0.57 (0.04,7.53)	ER+ECL						
0.30 (0.01,7.35)	0.37 (0.08,1.61)	0.65 (0.05,7.96)	ER+EL					
0.39 (0.02,8.24)	0.47 (0.12,1.89)	0.83 (0.08,8.32)	1.28 (0.34,4.87)	ECL				
0.57 (0.02,13.45)	0.69 (0.16,3.05)	1.22 (0.10,14.24)	1.88 (0.60,5.96)	1.47 (0.42,5.11)	EL			
0.61 (0.03,13.25)	0.74 (0.24,2.24)	1.30 (0.11,15.86)	2.02 (0.50,8.07)	1.58 (0.48,5.14)	1.07 (0.27,4.23)	SE		
0.10 (0.00,2.21)	**0.12 (0.03,0.47)**	0.21 (0.02,2.21)	**0.32 (0.11,0.98)**	**0.17 (0.07,0.45)**	**0.25 (0.09,0.72)**	**0.16 (0.05,0.55)**	ER	
1.19 (0.05,25.77)	1.44 (0.34,6.07)	2.55 (0.24,27.47)	**3.95 (1.16,13.45)**	**1.08 (1.99,9.55)**	**3.20 (2.69,6.33)**	1.96 (0.53,7.21)	**12.16 (4.22,35.07)**	BL

BL: blank group; ER: etch-and-rinse; SE: self-etch adhesive; EL: Er:YAG laser; ECL: Er,Cr:YSGG laser.

In terms of the incidence of adhesive failure mode, BL [OR = 0.41, 95% CI (0.17,0.99)], EL [OR = 3.70, 95% CI (1.71,7.99)] and ER+EL [OR = 3.79, 95% CI (1.53,6.52)] were more likely to occur compared to ER treatment (Table 3). Cohesive failure was more likely to occur in the ER group than in the EL group [OR = 0.21, 95% CI (0.08,0.58)] and ECL [OR = 0.29, 95% CI (0.11,0.75)] (Table 4). In terms of the mixed failure incidence, there was no statistical significance between the groups (Table 5).

**Table 3:** Odds ratio (OR) with 95% CI of adhesive failure mode

SE+EL							
1.45 (0.16,12.77)	ER+ECL						
0.49 (0.14,1.71)	0.34 (0.04,2.84)	ER+EL					
1.63 (0.51,5.19)	1.13 (0.16,7.83)	**3.30 (1.12,9.75)**	ECL				
0.51 (0.16,1.59)	0.35 (0.04,2.77)	1.02 (0.42,2.49)	**0.31 (0.11,0.84)**	EL			
1.29 (0.51,3.25)	0.89 (0.11,7.23)	2.61 (0.83,8.25)	0.79 (0.31,2.03)	2.55 (0.89,7.30)	SE		
1.87 (0.63,5.55)	1.29 (0.18,9.26)	**3.79 (1.53,9.39)**	1.15 (0.49,2.67)	**3.70 (1.71,7.99)**	1.45 (0.54,3.89)	ER	
0.76 (0.23,2.58)	0.53 (0.07,4.17)	1.55 (0.59,4.03)	0.47 (0.19,1.16)	1.51 (0.62,3.70)	0.59 (0.20,1.74)	**0.41 (0.17,0.99)**	BL

BL: blank group; ER: etch-and-rinse; SE: self-etch adhesive; EL: Er:YAG laser; ECL: Er,Cr:YSGG laser.

**Table 4:** Odds ratio (OR) with 95% CI of cohesive failure mode

SE+EL							
0.67 (0.05,8.31)	ER+ECL						
2.60 (0.55,12.22)	3.87 (0.34,43.74)	ER+EL					
2.47 (0.58,10.62)	3.68 (0.39,35.00)	0.95 (0.25,3.63)	ECL				
3.37 (0.74,15.39)	5.02 (0.46,54.27)	1.30 (0.38,4.45)	1.36 (0.38,4.89)	EL			
1.04 (0.32,3.31)	1.54 (0.14,17.59)	0.40 (0.09,1.68)	0.42 (0.12,1.45)	0.31 (0.08,1.25)	SE		
0.71 (0.19,2.69)	1.06 (0.12,9.36)	0.27 (0.09,0.84)	**0.29 (0.11,0.75)**	**0.21 (0.08,0.58)**	0.69 (0.20,2.30)	ER	
1.83 (0.42,8.03)	2.73 (0.26,28.33)	0.71 (0.22,2.28)	0.74 (0.25,2.17)	0.54 (0.18,1.67)	1.77 (0.48,6.51)	2.58 (0.97,6.90)	BL

BL: blank group; ER: etch-and-rinse; SE: self-etch adhesive; EL: Er:YAG laser; ECL: Er,Cr:YSGG laser.

**Table 5:** Odds ratio (OR) with 95% CI of mixed failure mode

SE+EL							
2.07 (0.09,48.75)	ER+ECL						
0.63 (0.11,3.73)	0.30 (0.01,6.60)	ER+EL					
0.38 (0.08,1.81)	0.18 (0.01,3.14)	0.61 (0.14,2.58)	ECL				
0.61 (0.12,3.01)	0.29 (0.01,5.78)	0.97 (0.28,3.31)	1.60 (0.47,5.38)	EL			
0.65 (0.17,2.39)	0.31 (0.01,6.57)	1.03 (0.20,5.22)	1.70 (0.48,5.97)	1.06 (0.26,4.35)	SE		
0.82 (0.18,3.63)	0.39 (0.02,6.86)	1.30 (0.37,4.54)	2.15 (0.81,5.68)	1.34 (0.50,3.60)	1.26 (0.34,4.71)	ER	
0.79 (0.15,4.22)	0.38 (0.02,7.66)	1.26 (0.36,4.40)	2.09 (0.62,7.08)	1.31 (0.44,3.85)	1.23 (0.28,5.33)	0.97 (0.34,2.81)	BL

BL: blank group; ER: etch-and-rinse; SE: self-etch adhesive; EL: Er:YAG laser; ECL: Er,Cr:YSGG laser.

The use of the 3M ESPE adhesives after acid etch-and-rinse significantly improved the shear bond strength and was superior to the other three adhesives, and SBS in the etch-and-rinse plus EL laser [SMD = 0.21, 95% CI (0.05,0.92)] group was greater than that of the EL laser alone [SMD = 0.06, 95% CI (0.02,0.21)] (Table 6).

**Table 6:** Standardized mean differences (SMDs) and 95% CI of bond strength of dentin (3M ESPE)

SE+ECL								
0.69 (0.03,17.60)	SE+EL							
0.14 (0.00,12.88)	0.20 (0.00,10.01)	ER+ECL						
0.67 (0.03,15.63)	0.97 (0.14,6.77)	4.78 (0.10,227.85)	ER+EL					
0.34 (0.02,6.73)	0.49 (0.05,4.66)	2.45 (0.05,125.21)	0.51 (0.06,4.12)	ECL				
2.40 (0.11,53.92)	3.46 (0.53,22.45)	17.12 (0.36,808.71)	3.58 (0.80,16.01)	6.99 (0.95,51.48)	EL			
0.43 (0.02,8.07)	0.62 (0.10,3.92)	3.05 (0.10,94.87)	0.64 (0.11,3.73)	1.25 (0.18,8.44)	0.18 (0.03,1.02)	SE		
0.14 (0.01,2.96)	0.21 (0.04,1.21)	1.03 (0.02,46.37)	**0.21 (0.05,0.92)**	0.42 (0.07,2.46)	**0.06 (0.02,0.21)**	0.34 (0.06,1.74)	ER	
1.90 (0.10,35.37)	2.74 (0.42,17.67)	13.55 (0.30,620.76)	2.83 (0.52,15.37)	5.53 (0.87,35.07)	0.79 (0.16,3.95)	4.44 (0.83,23.77)	**13.20 (3.06,56.90)**	BL

When using the Ivoclar Vivadent adhesives, the EL laser group significantly improved dentin bond strength compared to the blank group [SMD = 3. 20, 95% CI (1.19,8.62)]. The ECL laser group [SMD = 0.37, 95% CI (0.16,0.90)] had greater shear bond strength than the etch-and-rinse plus EL laser group [SMD = 0.25, 95% CI (0.07,0.85)] (Table 7).

**Table 7:** Standardized mean differences (SMDs) and 95% CI of bond strength of dentin (Ivoclar Vivadent)

SE+EL							
2.07 (0.09,48.75)	ER+ECL						
2.40 (0.52,11.11)	1.31 (0.28,6.06)	ER+EL					
3.15 (0.77,12.81)	0.87 (0.29,2.68)	0.67 (0.20,2.25)	ECL				
2.10 (0.67,6.63)	0.44 (0.12,1.66)	0.34 (0.11,1.05)	0.50 (0.19,1.31)	EL			
1.06 (0.30,3.69)	0.47 (0.12,1.81)	0.36 (0.10,1.30)	0.54 (0.23,1.26)	1.06 (0.35,3.20)	SE		
1.12 (0.36,3.56)	0.33 (0.10,1.12)	**0.25 (0.07,0.85)**	**0.37 (0.16,0.90)**	0.74 (0.30,1.83)	0.70 (0.24,2.02)	ER	
0.78 (0.23,2.70)	1.40 (0.42,4.75)	1.07 (0.32,3.61)	1.61 (0.68,3.82)	**3.20 (1.19,8.62)**	3.00 (0.98,9.24)	**4.30 (1.57,11.81)**	BL

There was no statistically significant difference in the bond strength of the self-etch adhesive plus EL laser group compared to the self-etch adhesive group when the Kuraray Dental adhesive was used (Table 8).

**Table 8:** Standardized mean differences (SMDs) and 95% CI of bond strength of dentin (Kuraray Dental)

SE+EL	1.06 (0.15,7.37)
0.94 (0.14,6.52)	SE

In the case of the adhesives by other manufacturers, the shear bond strength of the ECL laser group [SMD = 170.07, 95% CI (1.59,18184.10)] was greater than that of the etch-and-rinse plus EL laser group [SMD = 57.85, 95% CI (1.07, 3115.49)], and the etch-and-rinse group was higher than that of the self-etch adhesive group [SMD = 0.01, 95% CI (0.00, 0.45)] (Table 9).

**Table 9:** Standardized mean differences (SMDs) and 95% CI of bond strength of dentin (adhesives by “other” manufacturers)

SE+EL						
0.00 (0.00,0.24)	ER+EL					
0.00 (0.00,0.17)	0.34 (0.00,24.22)	ECL				
0.01 (0.00,1.43)	4.48 (0.12,168.52)	13.18 (0.24,727.82)	EL			
0.14 (0.00,13.98)	**57.85 (1.07,3115.49)**	**170.07 (1.59,18184.10)**	12.90 (0.34,496.14)	SE		
0.00 (0.00,0.14)	0.61 (0.02,16.19)	1.81 (0.09,37.47)	0.14 (0.01,2.83)	**0.01 (0.00,0.45)**	ER	
0.02 (0.00,3.10)	8.08 (0.19,339.54)	23.76 (0.71,795.04)	1.80 (0.07,46.85)	0.14 (0.00,9.80)	13.16 (0.70,247.92)	BL

### Probability Ranking

**Fig 3:**
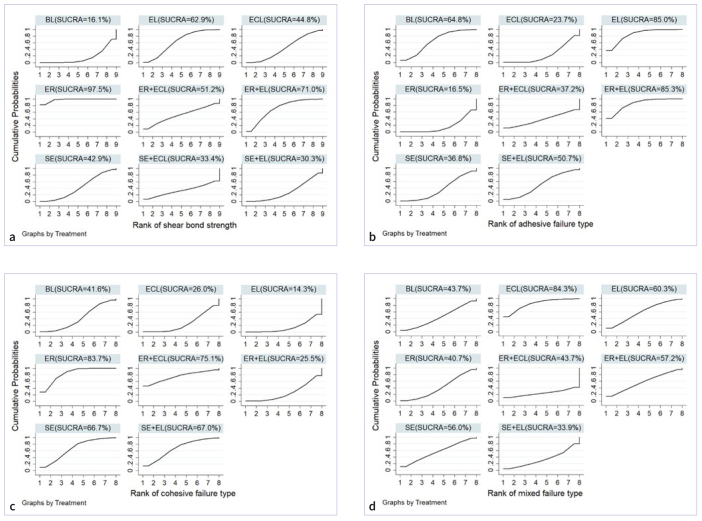
Probability ranking of all pretreatments. a. bond strength; b. adhesive failure mode; c. cohesive failure mode; d. mixed failure mode.

As shown in Fig 3a, the SUCRA probability ranking showed the ranking of the effect of different pretreatments on the bond strength of dentin. The SUCRA value indicated that ER (SUCRA = 97.5%) exhibited the highest likelihood of being the most effective pretreatment, the second most likely was ER+EL (71.0%), and the third EL (62.9%), followed by ECL (44.8%). Lower probabilities were found for SE (42.9%), SE+ECL (33.4%), and SE+EL (30.3%). BL (16.1%), with the lowest probability, ranked last.

Figure 3b shows the ranking of different pretreatments on the incidence of adhesive failure mode. The SUCRA value showed that ER+EL (SUCRA = 85.3%) had the highest probability of adhesive failure, followed by EL (85.0%), BL(64.8%), SE+EL (50.7%), ER+ECL (37.2%), SE (36.8%), and ECL (23.7%). ER (16.5%) had the lowest probability and ranked last.

Cohesive failure probability ranking results are shown in Fig 3c. According to the SUCRA value, ER (SUCRA = 83.7%) demonstrated the highest likelihood of experiencing cohesive failure, followed by ER+ECL (75.1%), SE (66.9%), SE+EL (66.5%), ER+EL (41.1%), BL (39.3%), and EL (26.2%). ECL (15.0%) had the lowest probability and ranked last.

Mixed failure probability ranking results are shown in Fig 3d. According to the SUCRA value, ECL (SUCRA = 84.4%) demonstrated the highest likelihood of cohesive failure, followed by EL (60.3%), ER+EL (57.2.0%) and SE (56.2%). BL (45.0%) and SE+EL (45.0%) had the same rate. ER (40.7%) and SE+EL (33.9%) had the second lowest and lowest probabilities.

### Publication Bias

**Supplementary Fig 2:**
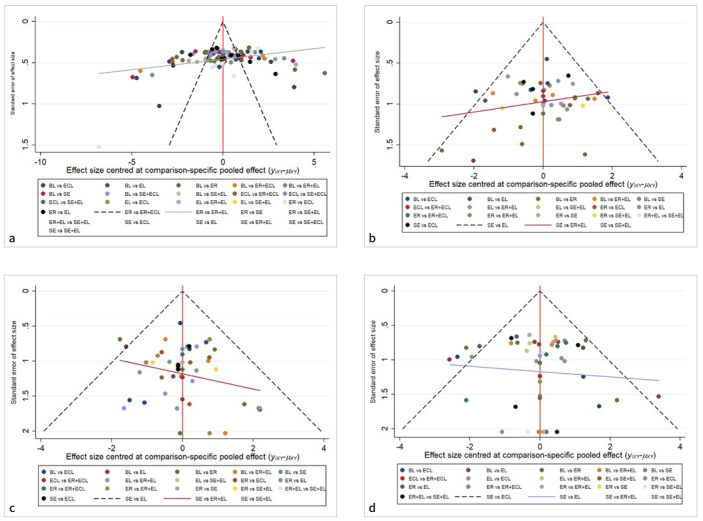
Funnel plots for different interventions (a. bond strength; b. adhesive failure mode; c. cohesive failure mode; d. mixed failure mode. BL: blank group; ER: etch-and-rinse; SE: self-etch adhesive; EL: Er:YAG laser; ECL: Er,Cr:YSGG laser.

A comparison-corrected funnel plot for each outcome indicator is shown in Supplementary Fig 2. Dentin SBS, adhesive failure mode, cohesive failure mode, and mixed failure mode scatter plots are located in the upper middle of the inverted triangle, and distribution is relatively symmetrical, suggesting a low risk of publication bias. However, some points were still located outside the funnel plot, suggesting a certain bias. In contrast, the results of Egger’s test showed p = 0.914 (statistical significance set at p < 0.05) for dentin SBS, p = 0.286 for adhesive failure mode, p = 0.694 for cohesive failure mode, and p = 0.633 for mixed failure mode, suggesting a small bias in the publication of the literature.

### Quality of the Evidence

The results of the CINeMA evidence summary of SBS showed that the overall quality of the 26 pairs of mixed comparisons and 10 pairs of indirect comparisons ranged between moderate and high quality (Supplementary Table 6). The CINeMA evidence summary of adhesive failure mode reveals that 19 pairs of mixed comparisons and nine pairs of indirect comparisons ranged between low and moderate quality (Supplementary Table 7). The CINeMA evidence summary of cohesive failure mode reveals that 19 pairs of mixed comparisons ranged between low and moderate quality, and 9 pairs of indirect comparisons were rated as very low-quality level of evidence (Supplementary Table 8). The CINeMA evidence summary of mixed failure mode reveals that 19 pairs of mixed and 9 pairs of indirect comparisons were rated as very low-quality evidence (Supplementary Table 9).

**Supplementary Table 6:** Confidence in estimates of bonding strength

Comparison	Number of studies	Within-study bias	Reporting bias	Indirectness	Imprecision	Heterogeneity	Incoherence	Confidence rating
Mixed evidence								
BL vs ECL	5	No concerns	Undetected	No concerns	No concerns	No concerns	No concerns	High
BL vs EL	12	Some concerns	Undetected	No concerns	No concerns	No concerns	No concerns	Moderate
BL vs ER	11	No concerns	Undetected	No concerns	No concerns	No concerns	Some concerns	Moderate
BL vs ER+ECL	1	Some concerns	Undetected	No concerns	No concerns	No concerns	No concerns	Moderate
BL vs ER+EL	7	No concerns	Undetected	No concerns	No concerns	No concerns	No concerns	High
BL vs SE	2	Some concerns	Undetected	No concerns	No concerns	No concerns	No concerns	Moderate
BL vs SE+ECL	1	Some concerns	Undetected	No concerns	No concerns	Some concerns	No concerns	Low
BL vs SE+EL	1	Some concerns	Undetected	Some concerns	No concerns	No concerns	No concerns	Low
ECL vs EL	1	Some concerns	Undetected	Some concerns	No concerns	No concerns	No concerns	Low
ECL vs ER	7	Some concerns	Undetected	No concerns	No concerns	No concerns	No concerns	High
ECL vs ER+ECL	2	Some concerns	Undetected	No concerns	No concerns	No concerns	No concerns	Moderate
ECL vs SE	5	Some concerns	Undetected	No concerns	No concerns	Some concerns	No concerns	Low
ECL vs SE+ECL	1	Some concerns	Undetected	No concerns	No concerns	Some concerns	No concerns	Low
ECL vs SE+EL	1	No concerns	Undetected	No concerns	No concerns	Some concerns	No concerns	High
EL vs ER	18	Some concerns	Undetected	No concerns	No concerns	No concerns	No concerns	Moderate
EL vs ER+EL	10	Some concerns	Undetected	No concerns	No concerns	No concerns	No concerns	Moderate
EL vs SE	1	No concerns	Undetected	No concerns	No concerns	No concerns	No concerns	High
EL vs SE+EL	1	Some concerns	Undetected	No concerns	No concerns	No concerns	No concerns	Moderate
ER vs ER+ECL	1	Some concerns	Undetected	No concerns	No concerns	Some concerns	No concerns	Low
ER vs ER+EL	13	Some concerns	Undetected	No concerns	No concerns	No concerns	No concerns	Moderate
ER vs SE	3	No concerns	Undetected	Some concerns	No concerns	No concerns	No concerns	High
ER vs SE+EL	3	No concerns	Undetected	No concerns	No concerns	No concerns	Some concerns	Moderate
ER+EL vs SE	3	Some concerns	Undetected	No concerns	No concerns	No concerns	No concerns	High
ER+EL vs SE+EL	3	No concerns	Undetected	No concerns	No concerns	Some concerns	No concerns	Moderate
SE vs SE+ECL	1	Some concerns	Undetected	No concerns	No concerns	No concerns	Some concerns	Low
SE vs SE+EL	10	Some concerns	Undetected	No concerns	No concerns	No concerns	No concerns	Moderate
Indirect evidence								
ECL vs ER+EL	/	Some concerns	Undetected	No concerns	No concerns	No concerns	No concerns	Moderate
EL vs ER+ECL	/	Some concerns	Undetected	No concerns	No concerns	No concerns	No concerns	High
EL vs SE+ECL	/	Some concerns	Undetected	Some concerns	No concerns	No concerns	No concerns	Moderate
ER vs SE+ECL	/	Some concerns	Undetected	Some concerns	No concerns	No concerns	No concerns	Moderate
ER+ECL vs ER+EL	/	Some concerns	Undetected	No concerns	No concerns	Some concerns	No concerns	Moderate
ER+ECL vs SE	/	Some concerns	Undetected	No concerns	No concerns	No concerns	No concerns	Moderate
ER+ECL vs SE+ECL	/	Some concerns	Undetected	No concerns	No concerns	No concerns	Some concerns	Low
ER+ECL vs SE+EL	/	No concerns	Undetected	No concerns	No concerns	No concerns	No concerns	High
ER+EL vs SE+ECL	/	No concerns	Undetected	No concerns	No concerns	Some concerns	Some concerns	Moderate
SE+ECL vs SE+EL	/	Some concerns	Undetected	No concerns	No concerns	Some concerns	No concerns	Moderate

1. The majority of evidence comes from studies that are determined to have a certain risk of bias, which warrants a downgrade by one level. 2. Confidence intervals crossing the boundaries of equivalence range result in a downgrade of one level. 3. Inconsistency between confidence intervals and prediction intervals regarding clinically important effects leads to a downgrade of one level. 4. Intersection of effect estimates for direct or indirect evidence from network meta-analysis with the minimal clinically important difference results in a downgrade of one level. BL: blank group; ER: etch-and-rinse; SE: self-etch adhesive; EL: Er:YAG laser; ECL: Er,Cr:YSGG laser.

**Supplementary Table 7:** Confidence in estimates of adhesive failure

Comparison	Number of studies	Within-study bias	Reporting bias	Indirectness	Imprecision	Heterogeneity	Incoherence	Confidence rating
Mixed evidence								
BL vs ECL	2	Some concerns	Undetected	No concerns	No concerns	No concerns	No concerns	Moderate
BL vs EL	7	Some concerns	Undetected	No concerns	No concerns	No concerns	Some concerns	Low
BL vs ER	6	No concerns	Undetected	No concerns	No concerns	No concerns	Some concerns	Moderate
BL vs ER+EL	5	Some concerns	Undetected	No concerns	No concerns	No concerns	Some concerns	Low
BL vs SE	1	No concerns	Undetected	No concerns	No concerns	No concerns	Some concerns	Moderate
ECL vs ER	6	Some concerns	Undetected	No concerns	No concerns	No concerns	No concerns	Moderate
ECL vs ER+ECL	1	Some concerns	Undetected	No concerns	No concerns	No concerns	No concerns	Moderate
ECL vs SE	3	Some concerns	Undetected	Some concerns	No concerns	No concerns	No concerns	Low
EL vs ER	8	Some concerns	Undetected	Some concerns	No concerns	No concerns	No concerns	Low
EL vs ER+EL	6	Some concerns	Undetected	No concerns	No concerns	No concerns	No concerns	Moderate
EL vs SE	1	Some concerns	Undetected	Some concerns	No concerns	No concerns	No concerns	Low
EL vs SE+EL	1	Some concerns	Undetected	Some concerns	No concerns	No concerns	No concerns	Low
ER vs ER+ECL	1	Some concerns	Undetected	No concerns	No concerns	Some concerns	No concerns	Low
ER vs ER+EL	5	No concerns	Undetected	No concerns	No concerns	Some concerns	No concerns	Moderate
ER vs SE	1	Some concerns	Undetected	No concerns	No concerns	No concerns	No concerns	Moderate
ER vs SE+EL	2	Some concerns	Undetected	Some concerns	No concerns	No concerns	No concerns	Low
ER+EL vs SE	1	No concerns	Undetected	No concerns	No concerns	No concerns	No concerns	High
ER+EL vs SE+EL	1	No concerns	Undetected	Some concerns	No concerns	No concerns	No concerns	Moderate
SE vs SE+EL	4	Some concerns	Undetected	Some concerns	No concerns	No concerns	No concerns	Low
Indirect evidence								
BL vs ER+ECL	/	Some concerns	Undetected	No concerns	Some concerns	No concerns	No concerns	Moderate
BL vs SE+EL	/	Some concerns	Undetected	Some concerns	No concerns	No concerns	No concerns	Low
ECL vs EL	/	Some concerns	Undetected	No concerns	No concerns	No concerns	No concerns	Moderate
ECL vs ER+EL	/	Some concerns	Undetected	No concerns	No concerns	Some concerns	No concerns	Moderate
ECL vs SE+EL	/	Some concerns	Undetected	No concerns	No concerns	No concerns	No concerns	Moderate
EL vs ER+ECL	/	Some concerns	Undetected	No concerns	Some concerns	No concerns	No concerns	Low
ER+ECL vs ER+EL	/	Some concerns	Undetected	No concerns	Some concerns	No concerns	No concerns	Low
ER+ECL vs SE	/	Some concerns	Undetected	No concerns	No concerns	Some concerns	No concerns	Low
ER+ECL vs SE+EL	/	Some concerns	Undetected	No concerns	No concerns	Some concerns	No concerns	Low

1. The majority of evidence comes from studies that are determined to have a certain risk of bias, which warrants a downgrade by one level. 2. Confidence intervals crossing the boundaries of equivalence range result in a downgrade of one level. 3. Inconsistency between confidence intervals and prediction intervals regarding clinically important effects leads to a downgrade of one level. 4. Intersection of effect estimates for direct or indirect evidence from network meta-analysis with the minimal clinically important difference results in a downgrade of one level. BL: blank group; ER: etch-and-rinse; SE: self-etch adhesive; EL: Er:YAG laser; ECL: Er,Cr:YSGG laser.

**Supplementary Table 8:** Confidence in estimates of cohesive failure

Comparison	Number of studies	Within-study bias	Reporting bias	Indirectness	Imprecision	Heterogeneity	Incoherence	Confidence rating
Mixed evidence								
BL vs ER	5	Some concerns	Undetected	No concerns	No concerns	No concerns	No concerns	Moderate
BL vs SE	1	No concerns	Undetected	No concerns	No concerns	No concerns	No concerns	High
BL vs EL	6	Some concerns	Some concerns	No concerns	No concerns	No concerns	No concerns	Low
BL vs ECL	2	Some concerns	Undetected	Some concerns	No concerns	No concerns	No concerns	Low
BL vs ER+EL	4	Some concerns	Undetected	No concerns	No concerns	No concerns	No concerns	Moderate
ER vs SE	1	No concerns	Undetected	No concerns	No concerns	No concerns	No concerns	High
ER vs EL	9	Some concerns	Undetected	No concerns	No concerns	No concerns	No concerns	Moderate
ER vs ECL	3	Some concerns	Undetected	No concerns	No concerns	No concerns	No concerns	Moderate
ER vs ER+EL	7	Some concerns	Some concerns	No concerns	No concerns	No concerns	No concerns	Low
ER vs ER+ECL	1	Some concerns	Undetected	Some concerns	No concerns	No concerns	No concerns	Low
ER vs SE+EL	1	Some concerns	Undetected	No concerns	No concerns	No concerns	No concerns	Moderate
SE vs EL	1	Some concerns	Undetected	No concerns	No concerns	No concerns	No concerns	Moderate
SE vs ECL	3	No concerns	Undetected	Some concerns	No concerns	No concerns	No concerns	Moderate
SE vs ER+EL	1	No concerns	Undetected	No concerns	No concerns	No concerns	No concerns	High
SE vs SE+EL	5	Some concerns	Some concerns	No concerns	No concerns	No concerns	No concerns	Low
EL vs ER+EL	5	Some concerns	Undetected	No concerns	No concerns	No concerns	No concerns	Moderate
EL vs SE+EL	1	Some concerns	Undetected	No concerns	No concerns	No concerns	No concerns	Moderate
ECL vs ER+ECL	1	Some concerns	Undetected	Some concerns	No concerns	No concerns	No concerns	Low
ER+EL vs SE+EL	1	No concerns	Undetected	No concerns	No concerns	No concerns	No concerns	High
Indirect evidence								
BL vs ER+ECL	/	Some concerns	Undetected	Some concerns	No concerns	No concerns	Major concerns	Very low
BL vs SE+EL	/	Some concerns	Some concerns	No concerns	No concerns	No concerns	Major concerns	Very low
SE vs ER+ECL	/	Some concerns	Some concerns	Some concerns	No concerns	No concerns	Major concerns	Very low
EL vs ECL	/	Some concerns	Undetected	No concerns	No concerns	No concerns	Major concerns	Very low
EL vs ER+ECL	/	Some concerns	Undetected	Some concerns	No concerns	No concerns	Major concerns	Very low
ECL vs ER+EL	/	Some concerns	Undetected	No concerns	No concerns	No concerns	Major concerns	Very low
ECL vs SE+EL	/	Some concerns	Some concerns	No concerns	No concerns	No concerns	Major concerns	Very low
ER+EL vs ER+ECL	/	Some concerns	Some concerns	Some concerns	No concerns	No concerns	Major concerns	Very low
ER+ECL vs SE+EL	/	Some concerns	Undetected	No concerns	No concerns	No concerns	Major concerns	Very low

1. The majority of evidence comes from studies that are determined to have a certain risk of bias, which warrants a downgrade by one level. 2. Confidence intervals crossing the boundaries of equivalence range result in a downgrade of one level. 3. Inconsistency between confidence intervals and prediction intervals regarding clinically important effects leads to a downgrade of one level. 4. Intersection of effect estimates for direct or indirect evidence from network meta-analysis with the minimal clinically important difference results in a downgrade of one level. BL: blank group; ER: etch-and-rinse; SE: self-etch adhesive; EL: Er:YAG laser; ECL: Er,Cr:YSGG laser.

**Supplementary Table 9:** Confidence in estimates of mixed failure

Comparison	Number of studies	Within-study bias	Reporting bias	Indirectness	Imprecision	Heterogeneity	Incoherence	Confidence rating
Mixed evidence								
BL vs ECL	3	Some concerns	Undetected	Some concerns	Some concerns	No concerns	Major concerns	Very low
BL vs EL	6	Some concerns	Undetected	Some concerns	No concerns	Major concerns	No concerns	Very low
BL vs ER	6	Some concerns	Undetected	Some concerns	No concerns	Major concerns	No concerns	Very low
BL vs ER+EL	4	Some concerns	Undetected	Some concerns	No concerns	Major concerns	No concerns	Very low
BL vs SE	1	Some concerns	Undetected	Some concerns	No concerns	Major concerns	Major concerns	Very low
ECL vs EL	1	Some concerns	Undetected	Some concerns	No concerns	Some concerns	No concerns	Very low
ECL vs ER	5	Some concerns	Undetected	No concerns	No concerns	Some concerns	No concerns	Low
ECL vs ER+ECL	1	Some concerns	Undetected	Major concerns	Some concerns	No concerns	No concerns	Very low
ECL vs SE	3	No concerns	Undetected	Some concerns	No concerns	Some concerns	No concerns	Very low
EL vs ER	8	Some concerns	Undetected	Some concerns	No concerns	Major concerns	Some concerns	Very low
EL vs ER+EL	4	Some concerns	Undetected	Some concerns	No concerns	Major concerns	No concerns	Very low
EL vs SE	1	Some concerns	Some concerns	Some concerns	No concerns	Major concerns	No concerns	Very low
EL vs SE+EL	1	Some concerns	Undetected	Some concerns	Some concerns	Some concerns	No concerns	Very low
ER vs ER+ECL	1	Some concerns	Some concerns	Major concerns	Some concerns	Some concerns	No concerns	Very low
ER vs ER+EL	4	Some concerns	Undetected	Some concerns	No concerns	Major concerns	No concerns	Very low
ER vs SE	1	Some concerns	Undetected	Some concerns	No concerns	Major concerns	No concerns	Very low
ER vs SE+EL	2	Some concerns	Undetected	Some concerns	No concerns	Major concerns	No concerns	Very low
ER+EL vs SE	1	Some concerns	Undetected	Some concerns	No concerns	Major concerns	No concerns	Very low
ER+EL vs SE+EL	1	Some concerns	Undetected	Some concerns	Some concerns	Some concerns	No concerns	Very low
SE vs SE+EL	3	Some concerns	Undetected	Some concerns	No concerns	Some concerns	No concerns	Very low
Indirect evidence								
BL vs ER+ECL	/	Some concerns	Some concerns	Major concerns	Major concerns	No concerns	Major concerns	Very low
BL vs SE+EL	/	Some concerns	Undetected	Some concerns	No concerns	Major concerns	Major concerns	Very low
ECL vs ER+EL	/	Some concerns	Some concerns	Some concerns	Some concerns	No concerns	Major concerns	Very low
ECL vs SE+EL	/	Some concerns	Undetected	Some concerns	Some concerns	No concerns	Major concerns	Very low
EL vs ER+ECL	/	Some concerns	Undetected	Major concerns	Major concerns	No concerns	Major concerns	Very low
ER+ECL vs ER+EL	/	Some concerns	Undetected	Some concerns	Major concerns	No concerns	Major concerns	Very low
ER+ECL vs SE	/	Some concerns	Undetected	Some concerns	Major concerns	No concerns	Major concerns	Very low
ER+ECL vs SE+EL	/	Some concerns	Undetected	Some concerns	Major concerns	No concerns	Major concerns	Very low

1. The majority of evidence comes from studies that are determined to have a certain risk of bias, which warrants a downgrade by one level. 2. Confidence intervals crossing the boundaries of equivalence range result in a downgrade of one level. 3. Inconsistency between confidence intervals and prediction intervals regarding clinically important effects leads to a downgrade of one level. 4. Intersection of effect estimates for direct or indirect evidence from network meta-analysis with the minimal clinically important difference results in a downgrade of one level. BL: blank group; ER: etch-and-rinse; SE: self-etch adhesive; EL: Er:YAG laser; ECL: Er,Cr:YSGG laser.

## Discussion

Effective dental bonding techniques are indispensible for success in restorative dentistry.^70^ Currently, acid etch-and-rinse and application of a self-etch adhesive are the commonly used methods for pretreating dentin surfaces.^73^ However, both techniques have been shown to possess drawbacks.^43,71^ Erbium laser has emerged as a promising tool in stomatology, particularly when applied to pretreat dentin surfaces.^3^ Nevertheless, the conclusion on whether erbium laser can be used to enhance the dentin SBS is contradictory.^4,27,39^ Therefore, in order to investigate whether erbium laser can enhance dentin SBS, we used an NMA. After a systematic and comprehensive analysis, we found that erbium lasers can indeed enhance dentin bond strength and may prove useful in guiding clinical dentin bonding protocols. Furthermore, the utilization of laser technology in conjunction with traditional acid-etching techniques can potentially augment dentin bonding effectiveness.

In this NMA, it was found that both modes of erbium lasers are effective in enhancing dentin bond strength as compared to the blank group. This phenomenon may be attributed to the micro-explosions induced by erbium laser irradiation, which vaporizes water and organic components within the tissue, consequently generating internal pressure until inorganic substances are explosively destroyed.^46^ Intertubular dentin, having a higher water content and lower mineral content than peritubular dentin, is selectively ablated by the laser, resulting in protruding dentinal tubules with a cuff-like appearance.^10^ This selective ablation potentially contributes to an increased adhesive area.^47^ Laser-treated dentin displays open tubules and a lack of smear layer, further promoting bonding. The enhanced adhesion to laser-treated dentin is believed to occur through the formation of resin tags and the seepage of adhesive resin into the micro-irregularities presented by demineralized dentin as a result of laser treatment.^15^

Based on the findings of the NMA, it can be concluded that acid etch-and-rinse combined with Er:YAG laser is the most effective treatment for improving dentin bond strength besides acid etch-and-rinse alone. The SUCRA value analysis supports this conclusion. Furthermore, the analysis of the impact of Er:YAG laser on dentin bond strength depending on different adhesives indicated that when using adhesive agents by 3M ESPE, the shear bond strength of acid etch-and-rinse combined with Er:YAG laser group was superior to that of the Er:YAG laser group alone. Although the erbium laser falls short of phosphoric acid etch-and-rinse in terms of improving the dentin SBS, it performs better in terms of safety, comfort, and reducing postoperative pain for patients.^40^ Notably, the dentin bond strength achieved with acid etch-and-rinse plus Er:YAG laser was higher than that achieved with Er:YAG laser alone. These results are consistent with the findings reported by Duun et al,^29^ Visuri et al,^74^ and Ceballos et al.^21^ Duun et al^29^ observed a significant improvement in SBS when dentin was acid etched after laser irradiation, compared to laser treatment alone. Similarly, Visuri et al^74^ found that combining acid etching with erbium laser pretreatment of dentin exposed more dentin tubules. This may be attributed to the ability of phosphoric acid to remove the surface scaling and flaking often observed on laser-ablated dentin surfaces. By clearing away the smear layer and opening up previously blocked dentin tubules, adhesive monomers can more easily penetrate the surface, facilitating bonding.

However, the SUCRA values also showed that acid etch-and-rinse combined with Er,Cr:YSGG laser ranked lower than Er,Cr:YSGG laser alone, indicating that the former may not be as effective as the latter. Interestingly, we observed similar results when using adhesive agents such as those by Ivoclar Vivadent or other manufacturers, where the shear bond strength of the Er,Cr:YSGG laser group alone was higher than that of the acid etch-and-rinse plus Er,Cr:YSGG laser group. Beer et al^14^ suggested that applying phosphoric acid etching after preparation with Er,Cr:YSGG laser could compromise the benefits of its ideal surface morphology. The acid not only dissolves the surface layer but also destroys the chimney-like formations of intertubular dentin and widens the orifices of the dentinal tubules. Moreover, using consecutive acid etching can result in unpredictable depths of the demineralization zone^76^ and lessening the diffusion depth of resin monomers.^78^ However, it should be noted that the conclusion of this analysis may have been influenced by the relatively small sample size.

The results of the SUCRA and NMA analysis suggest that the etch-and-rinse group had a lower incidence of adhesive failure and higher dentin bond strength than other groups. This finding is consistent with previous research by Garbui et al,^32^ which showed that lower adhesive strength often leads to increased adhesive failure. It should be noted that the acid etch-and-rinse combined with Er:YAG laser groups and Er,Cr:YSGG laser groups ranked higher in terms of SUCRA analysis, indicating lower bond strengths; however, in contrast, the NMA results of SBS showed that these groups had higher SBS. The observed discrepancy may be attributed to forming of weak, superficial areas and microcracks under the bonding interface due to Er:YAG laser irradiation of dentin. These structures can prevent effective penetration of the bonding resin into the dentin tubules and may be more susceptible to fracture under stress.^29^ This observation is supported by the findings of Guven et al.^37^ In conclusion, when evaluating dentin bonding efficacy, it is crucial to consider various factors, including the potential impact of treatment methods on the quality of dentin bonding.

Regarding cohesive failure, the SUCRA analysis indicated that the etch-and-rinse group had the highest incidence of cohesive failure, while NMA results also revealed the highest dentin bond strength in this group. These findings align with the view expressed by Almutairi et al.^8^ Moreover, the acid etch-and-rinse plus Er,Cr:YSGG laser group, the self-etch adhesive plus Er:YAG laser group, and the self-etch adhesive group also exhibited a higher incidence of cohesive failure and increased bond strength. However, the opposite trend was observed in the acid etch-and-rinse combined with Er:YAG group and Er:YAG laser groups, demonstrating a lower incidence of cohesive failure and higher dentin bond strength. This phenomenon may be attributed to the effects of laser irradiation on the composition and conformation of the organic matrix, which can hinder adhesive penetration and promote collagen degradation.^12^ Furthermore, the laser-treated dentin surface showed an etching pattern associated with structures resembling micro-fragmentation that could negatively impact the bonding of composite restorative materials.^26^ It is also worth noting that different laser parameters, such as pulse duration, energy, and material, used in various studies may contribute to the observed results. Thus, careful consideration of these factors is crucial when evaluating the efficacy of dentin bonding techniques.

In terms of mixed failure mode, Er,Cr:YSGG laser ranked first according to the SUCRA analysis, which is consistent with the findings of Al-Jeaidi et al.^1^ Several studies have suggested that factors such as the thermomechanical impact of Er,Cr:YSGG laser on the dentin surface, lateral forces, debonding protocol, and nature of conditioning pattern may contribute to this failure pattern.^7^ Er:YAG laser groups and Er,Cr:YSGG laser groups showed a high and similar incidence of mixed failure, which could be attributed to the non-uniform and heterogeneous etching pattern produced by the Er,Cr:YSGG laser.^88^ This results in areas between pulses that are not lased. It can be speculated that failure first occurs in the laser-ablated areas during the shear strength test, followed by adhesive or cohesive failure in the resin in areas not reached by the laser beam, where bonding to the dentin substrate is expected to be stronger. As a result, mixed failure is produced.^63^

We observed significant inconsistencies in the comparisons of BL vs ER, BL vs ECL, BL vs SE, and BL vs ER+EL for the three modes of bond failure. Further analysis suggests that these inconsistencies may be associated with various factors, including laser application parameters, adhesive, irradiation distance and duration, the spot size of the laser beam, water cooling, and the speed of the SBS test machine, as shown in Table 1.

Nahas et al^51^ pointed out that compared to the low-energy group (60 mJ), the high-energy erbium laser dentin pretreatment resulted in higher SBS. This may be because high-energy laser tends to melt the irradiated dentin, altering collagen fibers and causing their denaturation, sealing dentinal tubules, and preventing adhesive penetration into open tubules from forming resin tags.^31^ Cvkl et al^25^ observed that with the increase in energy of Er:YAG laser, the surface temperature of dentin rapidly rises, leading to localized overheating and resulting in phenomena such as melting, carbonization, and heat-induced damage, which in turn causes surface cracking of dentin and the formation of heat-induced damage layers that are difficult to remove by acid etching. Experimental results from Yaneva et al^80^ indicate that within Er:YAG laser frequencies of up to 50 Hz, laser energies of up to 200 mJ, and irradiation durations of up to 40 s, good efficiency in dentin cutting can be achieved while reducing the occurrence of heat-induced damage to dentin. Gurgan et al^36^ stated that when the power of the Er:YAG laser exceeds 2 W, it shows improved bond strength to dentin. Shirani et al^66^ suggested that as the distance of Er:YAG laser (30 Hz, 140 mJ) irradiation increases, the ablation produced on the surface of the irradiated tissue becomes more gentle and shallow, facilitating better adhesive penetration. As a result, the SBS between dentin and resin increases while the side effects of laser irradiation are reduced. Given the differences in laser parameters, bonding systems, laser irradiation distances and irradiation durations used in the different articles, the results of the above comparisons, which are subject to significant inconsistencies, should be viewed with caution.

This study has several limitations. For example, aging is one of the most important factors affecting dentin bonding. However, the literature reviewed here does not include the aspect of aging. Therefore, in future research, we will further investigate the effect of aging on dentin bonding.

## Conclusions

Based on the findings of this systematic review and network meta-analysis, the following conclusions were drawn:

- Both Er:YAG and Er,Cr:YSGG lasers improved dentin bond strength compared to the blank group, with Er:YAG laser being superior to Er,Cr:YSGG laser. Er:YAG laser combined with acid etch-and-rinse is the most effective treatment for enhancing dentin bond strength besides etch-and-rinse treatment alone.
- The use of 3M ESPE adhesives significantly increased the shear bond strength in the Er:YAG laser group, while the use of Ivoclar Vivadent adhesives or those of other manufacturers significantly increases the shear bond strength in the Er,Cr:YSGG group.
- Shear bond strength and mode of bond failure do not appear to be directly related. Despite certain limitations, our NMA provides significant insights into the clinical application of erbium laser in dentin bonding.

## Clinical relevance:

This is the first comprehensive and systematic meta-analysis to evaluate the influence of erbium laser and other dentin surface pretreatments on dentin SBS and failure modes. This NMA might provide valuable insights into formulating guidelines for dentin pretreatment with erbium laser.
